# Supplementary material for: Prefrontal mechanisms combining rewards and beliefs in human decision-making
Source: Nat Commun. 2019 Jan 17;10:301. doi: 10.1038/s41467-018-08121-w (PMC6336816; doi:10.1038/s41467-018-08121-w)
Supplement: Supplementary file 1 — Supplementary Information [file 41467_2018_8121_MOESM1_ESM.pdf]

Prefrontal mechanisms combining rewards and beliefs in human decision-making

Rouault et al.

Contents:

Supplementary Fig. 1
Supplementary Fig. 2
Supplementary Fig. 3
Supplementary Fig. 4
Supplementary Fig. 5
Supplementary Table 1
Supplementary Table 2
Supplementary Table 3
Supplementary Table 4
Supplementary Table 5
Supplementary Table 6
Supplementary Methods

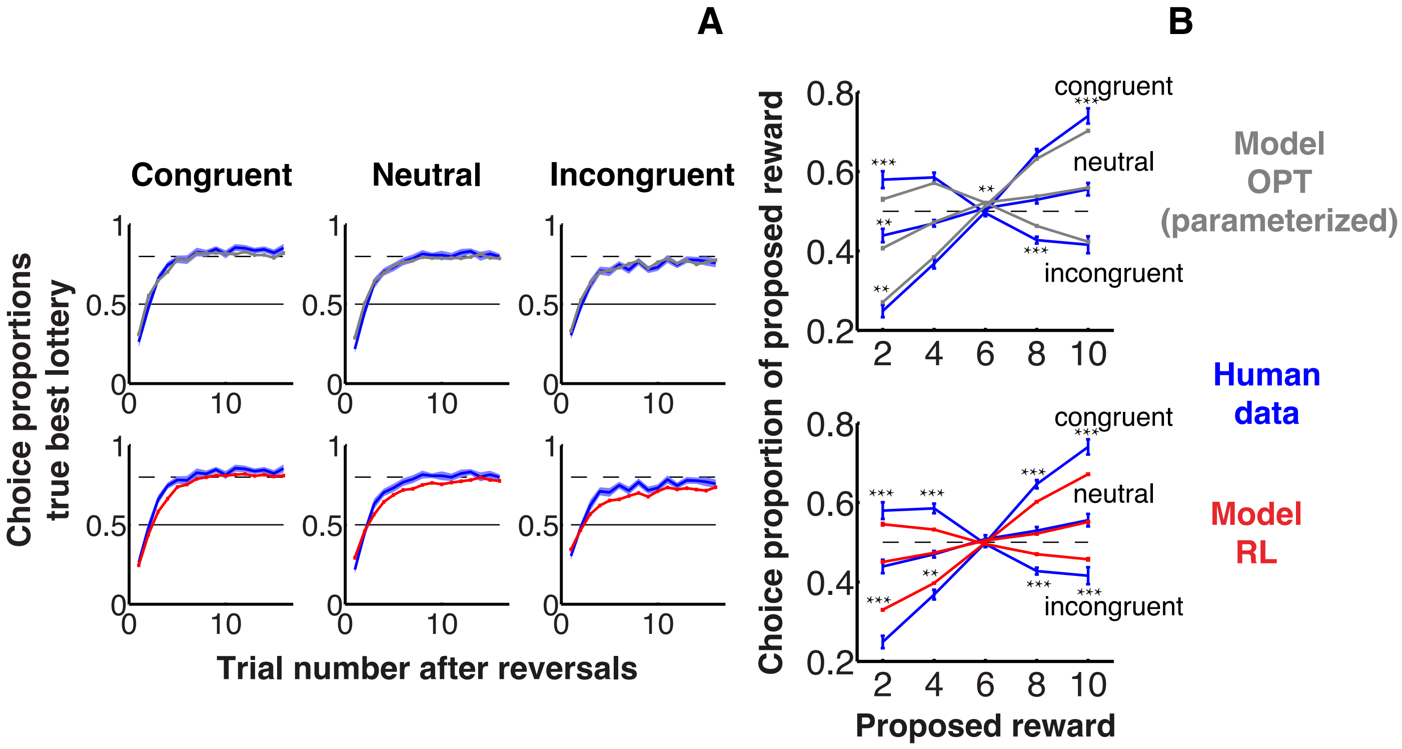

Supplementary Fig. 1 (related to Figs. 2 & 3). Simulations of fitted models RL (with proposed rewards) and OPT (parameterized).

Proportions of choosing true best bandits (maximizing reward frequency x proposed reward monetary value) according to trial number after reversals (**A**) and choice proportion of proposed rewards (**B**) for participants (blue, same data as in **Fig. 1**), model OPT (parameterized, gray) and model RL (including proposed rewards, red). Data are shown separately for the conditions congruent, neutral and incongruent. Parameterized model OPT corresponds to model DIST with no distortions on reward probabilities and values, and was fitted to human data with the same free parameters except the four parameters specifying distortions (see **Supplementary Table 1**). Model RL shown here included four free parameters: inverse temperature β, lapse rate ε, learning rate α, and contribution of proposed rewards to RL values φ at decision time. Error bars are s.e.m. across participants (N=22). Note that none of the models succeed in reproducing human data especially according to proposed rewards. In **B**, significant differences (T-tests) between model and human data are indicated (** p<0.01; ***p<0.001).

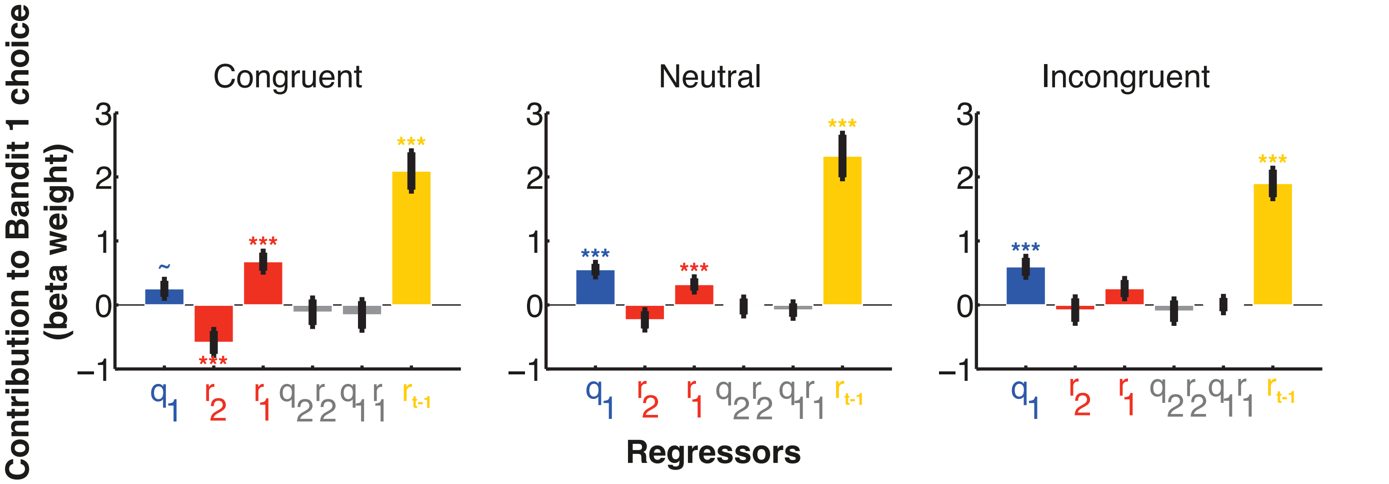

q_1 : reward frequency of bandit 1 (either 80% or 20%)

r_1 : proposed reward of bandit 1 (z-scored)

q_1r_1: reward expected values of bandit 1 (z-scored)

q_2 = 1- q_1 (so not included)

r_2 : proposed reward of bandit 2 (z-scored)

q_2r_2: reward expected values of bandit 2 (z-scored)

r_{t-1} : received reward in preceding trial (positive vs. negative if bandit 1 vs. 2 was chosen; z-scored)

Supplementary Fig.2 (related to Fig. 2). Influences of protocol parameters onto participants' choices. Logistic regression analyses in the congruent (left), neutral (center) and incongruent (right) conditions (full variance analyses so order of factors has no influence). Dependent variable *y* is bandit choices in every trial (*y*=1 if bandit 1 is chosen; *y*=0 otherwise). Independent variables are the protocol parameters: bandit reward frequencies (blue), proposed rewards (red), bandits' expected values (gray), and received reward in preceding trial (yellow). Note that bandits' expected values had no significant contribution (p>0.4) and indeed, removing these factors increased the fit in term of Bayesian Information Criteria (paired T-tests, $p<10^{-20}$). Consistent with model MIX, thus, proposed reward frequencies and values had only additive effects on participants' choices ,which further added up to the reward history (yellow regressor). Note also that relative to the neutral condition, the influence of proposed rewards increased in the congruent condition and decreased in the incongruent condition (interaction condition x proposed rewards: *F*=5.16, *p*=0.02). This indicates that consistent with model MIX, participants' choices were influenced by both the appetitive value and the information conveyed by proposed rewards about bandits' reward frequencies. In the congruent condition, indeed, rewards were biased towards higher values for the more frequently rewarded bandit (80%), so that the appetitive value and the information conveyed by proposed rewards about the more frequently rewarded bandit had congruent influences on choices. In the incongruent condition, by contrast rewards were biaised towards lower values for the more frequently rewarded bandit, so that the appetitive values and the information conveyed by proposed rewards about the more frequently rewarded bandid had opposite influences on choices. ~p=0.057; **p<0.01; ***p<0.001 (T-tests). Error bars are s.e.m. across participants (N=22).

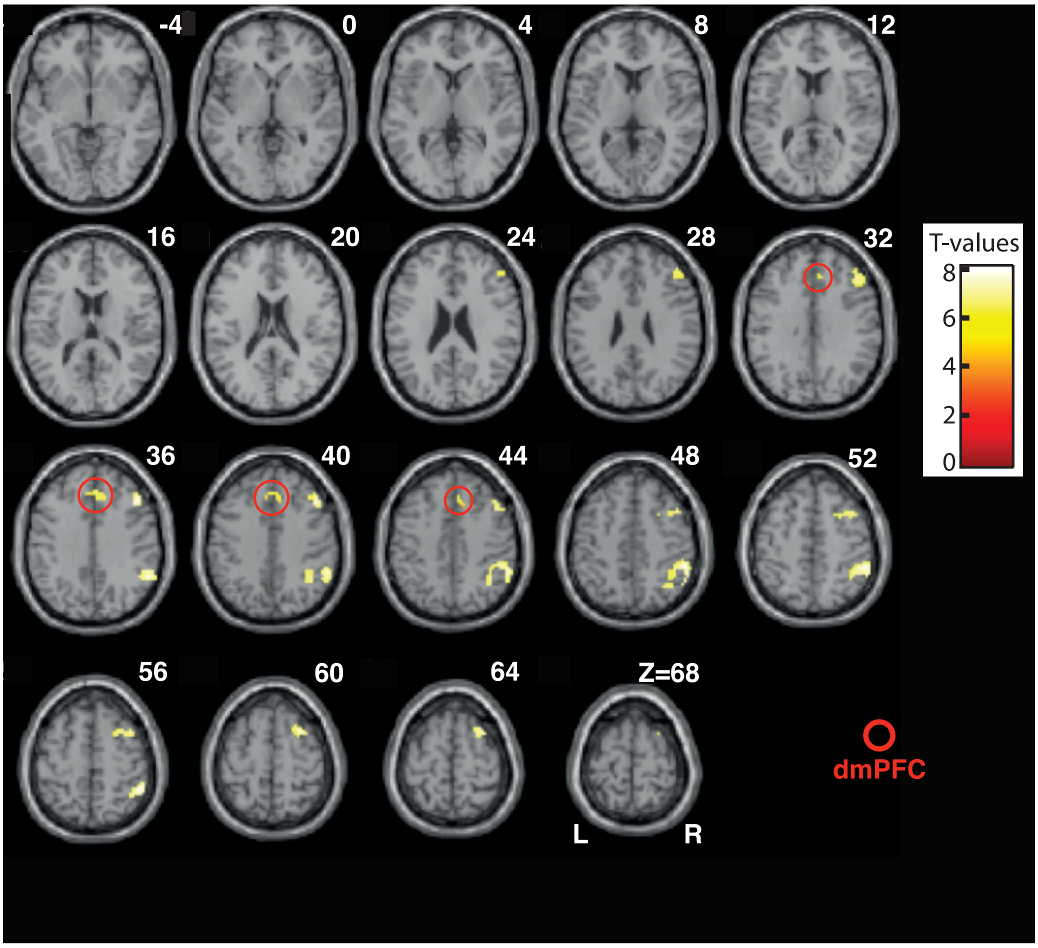

Supplementary Fig. 3 (related to Fig. 4). Activation map associated with decision entropy when factoring out reaction times. Yellow, brain activations associated with an inverted U-shape function of decision variable $(1)\gamma(v_1-v_2)+\gamma(B_1-B_2)$ centered on zero from model MIX overlaid on a canonical single-subject anatomical template (neurological convention). Axial slices are indexed by their MNI Z coordinate. Red circles highlight dorsomedial prefrontal activations (dmPFC, Broadman's area 32). Activations are those computed in **Fig. 4** and listed in **supplementary Table 3**, which remained significant ($p<0.001$, uncorr. T-tests) after factoring out reaction times. Note that compared to **Fig. 4**, only dmPFC, right prefrontal and parietal activations remained significant. In particular, dmPFC activations were identical to those found in **Fig. 4**: same extension, same activation peak (MNI coordinates: 6, 32, 34; T=6.17). N=21 participants.

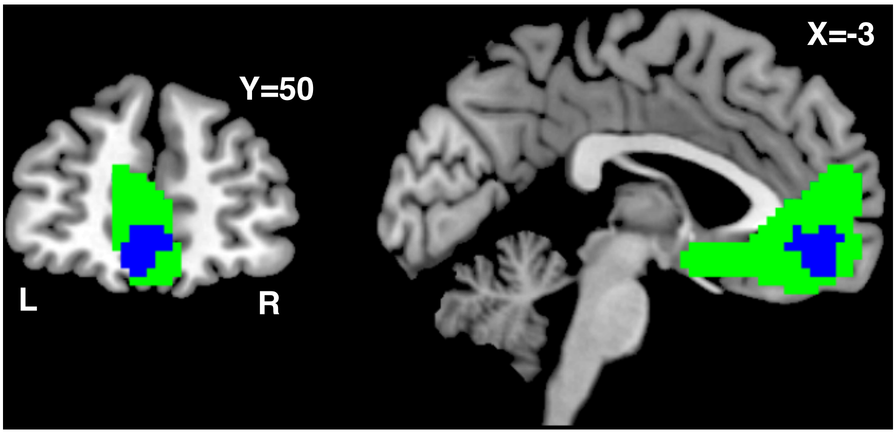

Supplementary Fig. 4. Psycho-Physiological Interactions associated with relative chosen beliefs with the dmPFC as seed region. Green, vmPFC activations encoding relative beliefs, irrespective of choice computations, i.e. associated with $(B_{chosen}\text{-}B_{unchosen})^2$ (same data as in **Fig. 7**). Blue, significant Psycho-Physiological Interactions (PPIs) within these vmPFC activations: the correlation between dmPFC and vmPFC activity in the blue voxels decreased when relative chosen beliefs increased (T-tests, $p<0.05$, cluster size: 95 voxels or 2.6 cm^3; peak: $T=2.92$, $p=0.004$, MNI coordinate: $x=-6$, $y=50$, $z=-11$). Activations are superimposed on coronal and sagittal anatomical slices from MNI template (indexed by their MNI coordinate, neurological convention). PPIs were analyzed using the standard SPM8 method. The seed region (ROI) was the dmPFC activation reflecting choice computations shown in **Fig. 5**. Time series of ROI activity were extracted from single-subject activation peaks (associated with the negative linear effect of relative chosen beliefs) within this ROI and adjusted for factors of no interest (scanning runs, movements, no response trials). ROI activity regressors and PPI regressors modeling the interaction between ROI activity and relative chosen beliefs $(B_{chosen}\text{-}B_{unchosen})$ were then formed and added to GLM#2 (see Methods), which included all regressors of interest (relative chosen beliefs, relative chosen utilities and their quadratic expansion) in a full variance analysis. N=21 participants.

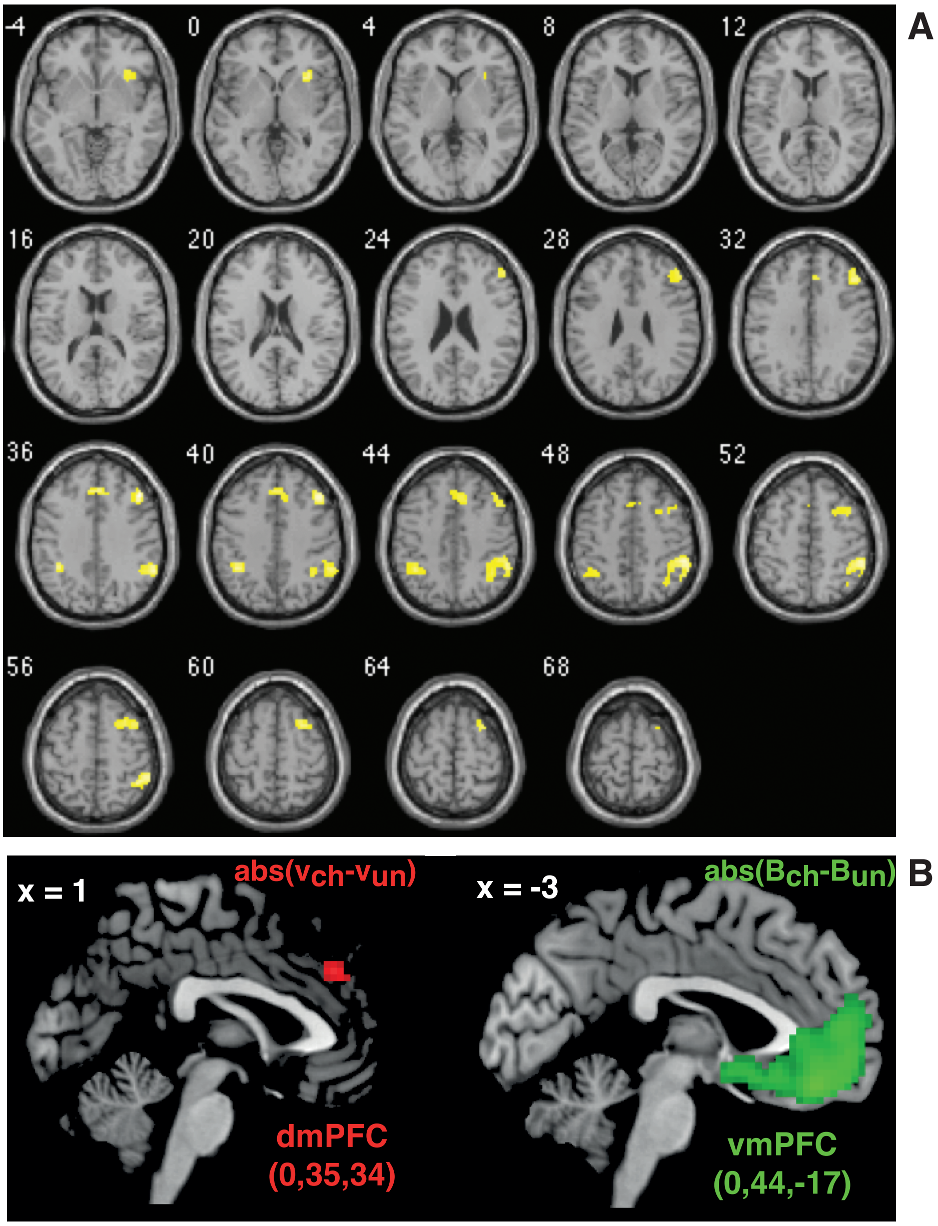

Supplementary Fig. 5 (related to Figs 4 & 7). GLM analyses with modulus operator rather than quadratic expansion.
A, same GLM analysis as in **Fig. 4** with absolute values of decision variable $(1\text{-}\omega)(v_1\text{-}v_2)+\omega(B_1\text{-}B_2)$ rather than its quadratic expansion. Note that activations are similar to those in **Fig. 4**. dmPFC activations are virtually identical to those in **Fig. 4**. Voxel-wise threshold $p<0.05$, FWE-corrected for multiple comparisons over the whole brain . Cluster-wise threshold $p<0.05$. **B**, same GLM analysis as in **Fig. 7** with absolute values of relative normalized utilities $(v_1\text{-}v_2)$ and affective beliefs $(B_1\text{-}B_2)$ rather than their quadratic expansion. Number in brackets are MNI coordinates of activation peaks. Note that dmPFC and vmPFC activations along with activation peaks are similar to those in **Fig. 7**. Voxel-wise threshold $p<0.05$ FWE-corrected for multiple comparisons over the frontal lobes (MNI coordinate $Y>0$). Cluster-wise threshold $p<0.05$. N=21 participants.

Supplementary Table 1 (related to Fig. 2).

Model DIST's best fitting parameters. Mean and S.E.M. across subjects (N=22).

Parameters	Description	Mean	S.E.M.
β	Inverse temperature (softmax)	43.0	7.9
ε	Lapses rate	0.04	0.02
ν	Volatility	0.11	0.03
q_M=1-q_m	Reward frequency (true value=0.8)	0.88	0.03
γ_c	Reward bias slope, congruent condition	0.080	0.050
γ_n	Reward bias slope, neutral condition	0.006	0.005
γ_i	Reward bias slope, incongruent condition	-0.045	0.050
η_p	Distortion slope for probabilities	3.11	0.44
P_0_p	Distortion fixed point for probabilities	0.23	0.06
η_r	Distortion slope on rewards	1.85	0.43
P_0_r	Distortion fixed point for rewards	0.34	0.09

Supplementary Table 2 (related to Fig. 3).
Model MIX best fitting parameters. Mean and S.E.M. across subjects (N=22).

Parameters	Description	Mean	S.E.M.
β	Inverse temperature (softmax)	54.9	9.6
ε	Lapses rate	0.02	0.01
ν	Volatility	0.16	0.03
$q_{M=}1\text{-}q_m$	Reward frequency (true value=0.8)	0.76	0.04
γ_c	Reward bias slope, condition congruent	0.10	0.06
γ_n	Reward bias slope, condition neutral	0.05	0.04
γ_i	Reward bias slope, condition incongruent	-0.10	0.06
α	Learning rate in RL	0.63	0.08
$1-\varphi$	RL contribution to utilities	0.80	0.07
ω	weight mixing utilities and beliefs	0.69	0.06

Supplementary Table 3. GLM#1 (related to Fig. 4)
Brain activations (whole brain analysis, $p<0.05$ voxel-wise, corrected for multiple comparison, cluster-wise threshold: $p<0.05$; voxel size: 3 mm cubic) associated with *decision entropy*. N=21 participants.

**Regions**		**Abbrev.**	**Peak MNI coordinates**			**T-values**	**Size (cm^3)**
			**x**	**y**	**z**		
Negative quadratic effect of decision variable: $-[(1-\omega)(v_1-v_2)+\omega(B_1-B_2)]^2$							
Lateral prefrontal cortex (BA9 to 8)	right	laPFC	45	26	37	8.97	4.3
Lateral prefrontal cortex (BA 9)	left	laPFC	-48	29	31	8.04	0.5
Dorsomedial prefrontal cortex (BA 32, dACC)		dmPFC	6	32	34	7.63	1.1
Polar prefrontal cortex (BA 10)	right	poPFC	39	59	10	7.65	0.5
Insular cortex (anterior)	right	Ins	30	20	1	7.58	0.6
Inferior parietal lobule (BA 40)	right	IPL	54	-40	52	8.60	5.4
Inferior parietal lobule (BA 40)	left	IPL	-39	-46	40	7.31	0.7
Precuneus (BA 7)		prCU	9	-67	43	7.74	0.8

Supplementary Table 4. GLM#2 (related to Figs. 5 & 7)
Frontal lobe activations ($p<0.05$ voxel-wise, corrected for multiple comparison over the frontal lobes: MNI coordinate Y>0; cluster-wise threshold: $p<0.05$; voxel size: 3 mm cubic) associated with affective state beliefs and normalized utilities. N=21 participants.

**Regions**	**Abbrev.**	**Peak MNI coordinates**			**T-values**	**Size (cm^3)**
		**x**	**y**	**z**		
Joint negative linear effects of relative chosen beliefs -(B_{ch}-B_{un}) and normalized utilities -(v_{ch}-v_{un}) with no interactions between these factors (p>0.05, uncorr. T-tests)						
Dorsomedial prefrontal cortex (dACC)	dmPFC					11.9
$-(B_{ch}-B_{un})$		-6	32	37	7.05	
$-(v_{ch}-v_{un})$		3	26	37	6.28	
Right premotor cortex	PM					1.5
$-(B_{ch}-B_{un})$		27	5	58	5.16	
$-(v_{ch}-v_{un})$		27	5	67	4.60	
Right Insula	Ins					6.0
$-(B_{ch}-B_{un})$		30	26	-2	8.83	
$-(v_{ch}-v_{un})$		30	33	-8	4.47	
Positive quadratic effect of relative state beliefs $(B_{ch}-B_{un})^2$						
Ventromedial prefrontal cortex	vmPFC	-3	41	-14	4.19	25.5
Positive quadratic effect of relative normalized utilities $(v_{ch}-v_{un})^2$						
Dorsomedial prefrontal cortex (dACC)	dmPFC	3	26	37	3.82	2.4
Positive linear effect of relative chosen normalized utilities $v_{ch}-v_{un}$						
Ventromedial prefrontal cortex	vmPFC	0	26	-14	5.08	13.9

Supplementary Table 5. GLM#3 (related to Fig. 8)
Frontal lobe activations (p<0.05 voxel-wise, corrected for multiple comparison over the frontal lobes: Y>0 cluster-wise threshold: p<0.05; voxel sizes: 3 mm cubic) associated with value components of normalized utilities. N=21 participants.

**Regions**		**Abbrev.**	**Peak MNI coordinates**			**T-values**	**Size (cm^3)**
			**x**	**y**	**z**		
Negative linear effect of relative chosen RL-values -$(V_{ch}^{RL} - V_{un}^{RL})$							
Insula (anterior)	right	Ins	42	27	7	4.49	2.9
Premotor cortex (BA 6)	right	PM	38	8	61	3.54	2.0
Negative linear effect of relative chosen proposed rewards -$(V_{ch}^{pr} - V_{un}^{pr})$							
None							
Positive quadratic effect of relative RL-values $(V_{ch}^{RL} - V_{un}^{RL})^2$							
Lateral orbitofrontal cortex (BA 11)	right	laOFC	39	35	-17	4.75	2.9
Lateral orbitofrontal cortex (BA 11)	left	laOFC	-39	35	-17	3.92	0.9*
Positive quadratic effect of relative proposed rewards $(V_{ch}^{pr} - V_{un}^{pr})^2$							
None							
Positive linear effect of relative chosen RL-values $V_{ch}^{RL} - V_{un}^{RL}$							
None							
Positive linear effect of relative chosen proposed rewards $V_{ch}^{pr} - V_{un}^{pr}$							
Ventromedial prefrontal cortex		vmPFC	3	38	-11	3.54	21
Dorsolateral prefrontal cortex (BA 8)	left	dlPFC	-24	23	43	3.58	9.4

* subthreshold activation extension, but mentioned here because in the exact same location as its right homolog.

Supplementary Table 6. Fitting values for main tested models*.

Model (# free parameters)	LLH†	BIC†	AIC†
MIX (10)	-400.9 (23.2)	**872.1**‡ (46.4)	**821.8**‡ (46.4)
DIST (11)	-405.3 (22.6)	888.0 (45.1)	832.6 (45.2)
DIST+RL (13)	-399.8 (22.5)	890.4 (44.9)	824.9 (44.9)
Parameterized OPT (7)	-438.9 (21.8)	926.9 (43.5)	891.7 (43.5)
Parameterized OPT+RL (9)	-413.1 (22.9)	896.5 (45.8)	846.2 (45.8)
RL+proposed rewards (4)	-469.9 (23.7)	968.0 (47.4)	947.9 (47.4)

*Mean (sem) across participants (N=22).
† LLH: loglikelihood. BIC: Bayesian Inform. Criter. AIC: Akaike Inform. Criter.
‡ Best fitting values.

Supplementary methods

Note that the linear and quadratic expansion regressor associated with a variable X_{chosen}-$X_{unchosen}$ driving choices share some variance as choices more frequently sample positive values of X_{chosen}-$X_{unchosen}$ (sampling bias). However, the linear and quadratic function mathematically form orthogonal regressors, so that the share variance captures only the effects due to the sampling bias, while the linear and quadratic regressor entirely capture the linear and quadratic component, respectively. In a full variance regression analysis including multiple regressors, the shared variance is assigned to the residuals, while the variance attributed to each regressor capture the specific contribution of each regressor to activations. Accordingly, the full variance regression analysis properly assigns the sampling bias to the residuals, while the variance attributed to the linear and quadratic regressor entirely captures the linear and quadratic component in activations, respectively.

For that reason, all results reported in the paper were based on full variance regression analyses. As indicated in the main text, the presence of a negative linear effect in activations reflects selection processes based on variable X, while the presence of a positive quadratic effect (with a quadratic regressor properly centered on zero corresponding to maximal coding entropy) reflects the encoding of variable X, irrespective of selection processes (see also [1]). The presence of both effects in the same region has a direct interpretation: the region is involved both in encoding variable X irrespective of choices and in computing choices based on variable X. Note that one might alternatively consider that this region exhibits activations varying as a "pure" quadratic function of variable X centered on a non-zero value (indeed, $X^2 - aX = (X - a/2)^2 + \text{constant}$). We dismissed this alternative interpretation because a non-zero value $a/2$ has no significance in the present protocol (moreover, Duverne & Koechlin [1] provided empirical evidence ruling out this alternative interpretation: they found that only the zero-centered quadratic effect remained present when choices were independent of variable X). As the dmPFC was found to exhibit both effects associated with normalized utilities (**Figs. 5 & 7**), we thus concluded that the dmPFC both encodes normalized utilities and computes choices based on these normalized utilities (along with state beliefs as the dmPFC also showed a negative linear effect associated with beliefs, **Figs. 5 & 7**). This interpretation is further in agreement with the inverted quadratic effect observed in the dmPFC and associated with the decision variable linearly combining utilities and beliefs.

In order to replicate previous studies investigating only linear effects, we also conducted regression analyses whereby quadratic regressors were projected/orthogonalized onto linear regressors. This operation arbitrary assigns the shared variance to linear regressors rather than to residuals. The variance attributed to linear regressors is then identical to that obtained from a regression analysis including only linear regressors (while the variance attributed to quadratic regressors remains identical to that obtained in the full variance regression analysis). In that case, however, the variance attributed to linear regressors mixes both true linear effects *and the effects of sampling biases*.

For normalized utilities, the sampling bias was actually small because according to model MIX, normalized utilities had a small contribution to choices. Consistently, this regression analysis provided virtually the same results regarding normalized

utilities as the full variance regression analysis reported in the paper (**GLM#2**): in particular, no additional activations exhibited linear effects associated with relative chosen utilities. For state beliefs, by contrast, the sampling bias was large because state beliefs predominantly contribute to choices. As expected, thus, the control analysis showed the same activations as the full variance analysis *plus* additional activations associated with the linear regressor $X = B_{\text{chosen}} - B_{unchosen}$ (see **Discussion**). These additional "linear" activations were found in the vmPFC (in addition to the quadratic effect of relative state beliefs found in the full variance analysis). As these "linear" activations were not found in the full variance analysis, we can conclude that they reflect the sampling bias rather than a genuine linear component in activations.

Supplementary references

[1] Duverne S, Koechlin E. Rewards and Cognitive Control in the Human Prefrontal Cortex. Cereb Cortex 27, 5024-5039 (2017)
